# Musculoskeletal Adverse Events Associated with Adjuvant Aromatase Inhibitors

**DOI:** 10.1155/2010/654348

**Published:** 2010-08-24

**Authors:** Qamar J. Khan, Anne P. O'Dea, Priyanka Sharma

**Affiliations:** The University of Kansas Medical Center, 3901 Rainbow Boulevard., Kansas City, KS 66160, USA

## Abstract

Musculoskeletal symptoms including arthralgia and myalgia occur frequently in aging women, particularly during the transition to menopause, when plasma estrogens precipitously decline. In postmenopausal women (PMW) with breast cancer, third-generation aromatase inhibitors (AIs) as adjuvant hormonal therapy have proven to be more effective, and to have a more predictable side effect profile, than tamoxifen. However, AIs further reduce plasma estrogens in PMW, exacerbating musculoskeletal symptoms. Clinical trial data have shown significantly higher incidences of arthralgia and myalgia with AIs compared with women on tamoxifen or placebo. Symptoms may be severe enough to significantly affect quality of life; musculoskeletal symptoms are a frequent reason for discontinuing therapy. In many cases, symptoms can be effectively managed with oral analgesics or other strategies. Early recognition and effective management of musculoskeletal symptoms can help maximize treatment compliance, enabling patients to derive optimal benefit from therapy in terms of preventing recurrence.

## 1. Introduction

The third-generation aromatase inhibitors (AIs) have proven superior to tamoxifen when used as adjuvant hormonal therapy in postmenopausal women with hormone receptor positive (HR+) early breast cancer. Major trials have established that AIs improve disease-free survival (DFS) when used either as initial adjuvant therapy or as a switch/sequential option for patients completing 2 or more years of prior tamoxifen treatment [1–4], and the use of AIs as adjuvant endocrine therapy for postmenopausal women with HR+ breast cancer has been steadily increasing [5]. AIs are also an attractive alternative to tamoxifen because of their adverse event (AE) profile, which closely mimics symptoms commonly associated with the menopause-related decline in estrogen level while lacking the rare, yet serious, AEs associated with tamoxifen (e.g., endometrial cancer and venous thromboembolism) [6]. The safety of adjuvant anastrozole, letrozole, and exemestane has been established in major clinical studies including the arimidex tamoxifen alone or in combination (ATAC) trial, the breast international group 1-98 (BIG 1-98) trial, and the intergroup exemestane study (IES), respectively [3, 4, 7, 8]. While these trials differ in study design, patient population, and AE reporting criteria, they have generally shown similar safety profiles, with a predominance of mild-to-moderate menopause-related symptoms such as loss of bone density, musculoskeletal symptoms such as arthralgias, myalgias, and hot flashes. Arthralgias and myalgias in particular are among the most commonly reported musculoskeletal AEs [9–11] and are an important cause of treatment discontinuation [11, 12]. In this paper, we examine the incidence of musculoskeletal side effects with the different AIs, with an emphasis on reducing and managing these AEs in postmenopausal women receiving adjuvant AI therapy.

## 2. The Syndrome of AI-Associated Arthralgia and Myalgia

While the clinical presentation may vary considerably, arthralgia typically includes symmetrical pain or stiffness in the joints that is not associated with inflammatory processes and the joint destruction of arthritis [13]. Soreness in the hands, knees, hips, lower back, shoulders and/or feet, early morning stiffness, and difficulty sleeping may be present, and patients may experience symptoms such as rings not fitting as they once did, because of mild thickening of soft tissues [13]. Symptoms of arthralgia and myalgia may also include an impaired ability to completely close or stretch the hand and/or fingers and difficulty performing daily activities such as dressing, driving, or typing [14]. Another cue suggestive of arthralgia is patients' “feeling like they have aged suddenly” upon squeezing or flexing the affected joints [13]. A recent prospective study evaluating changes in the hands and wrists of patients taking AIs (*N* = 12) or tamoxifen (*N* = 5) found that after 6 months, AI users were >2-fold more likely to have decreased grip strength compared with tamoxifen users (relative risk [RR], 2.08), and >3.5 times more likely than tamoxifen users to have magnetic resonance imaging (MRI)-assessed worsening of tenosynovial changes (RR, 3.67) [15]. Worsening of tenosynovial changes was found to be strongly related to a higher decrease in grip strength (Spearman correlation, −0.64; *P* = .0074). These findings suggest that AI users are more likely than tamoxifen users to have new-onset or worsening of preexisting musculoskeletal symptoms, and that these symptoms could be correlated with functional impairment and objectively assessed MRI changes [15]. Another recent study examined AI-related arthralgia in women receiving AIs (*n* = 92) and a control group not receiving hormonal therapy (*n* = 28) [16]. Investigators found a 33% incidence of AI-related new-onset or worsening arthralgia; the most commonly affected joints were the knee (70%), wrist (70%), and small joints in the hand (63%). Patients receiving AIs with arthralgia had more joint and tendon effusions (69% versus 42%; *P* < .05) and electrophysiologic findings consistent with carpal tunnel syndrome (46% versus 20%; *P* < .05) compared to those without arthralgia, and those receiving AIs had thicker tendons compared to those never receiving AIs (*P* < .001) [16]. However, there was no evidence of an autoimmune or inflammatory process associated with AI use, and the authors acknowledged that some patients with AI-related arthralgia had no evidence of tenosynovial or electrophysiologic changes; the latter findings would seem to preclude a straightforward association between these changes and the symptoms of arthralgia in patients taking AIs.

## 3. Risk Factors

Menopausal status appears to be an important risk factor for musculoskeletal symptoms in women. In a study of the natural history of menopausal symptoms in women aged 40−55 years (*N* = 237), joint/muscle pain was reported by over half (57%) [17]. About one third (28.7%) of the women were perimenopausal, and these women showed the highest incidence of joint/muscle pain (67.6%) compared with pre- (54%) or postmenopausal women (48.8%) [17]. Another community-based study of healthy women found that postmenopausal women (*n* = 263), compared with premenopausal (*n* = 734) or perimenopausal women (*n* = 363), experienced a significantly higher incidence of arthralgia (38.6% versus 29.8% versus 35.2%, resp.; *P* = .028) [18]. This study also found that postmenopausal women had significantly lower scores on multiple measures of a quality of life (QoL) questionnaire (Short Form 36) compared with premenopausal women [18]. Another study, in Sweden, found that shoulder, arm, leg, and/or back pain was more common in women than in men among individuals between 20 and 64 years of age [19]. This study also showed that the percent of female subjects with musculoskeletal pain increased with increasing body mass index (BMI) and decreased with increasing physical activity. These findings highlight the importance of menopausal status, weight, and physical activity on the risk for arthralgia in women.

Risk factors for joint symptoms were examined in patients with breast cancer enrolled in the ATAC trial, which compared initial adjuvant treatment with anastrozole relative to tamoxifen. Only women who did not report joint symptoms at randomization were included in the analysis (anastrozole, *n* = 2,698; tamoxifen, *n* = 2,735), so as to evaluate new-onset (and likely treatment-related) joint symptoms [20]. The results showed higher overall joint symptoms in the anastrozole-treated group versus the tamoxifen-treated group (35.2% versus 30.3%, resp.; odds ratio [OR], 1.25; 95% confidence interval [CI], 1.11–1.40), with a similar distribution of severity between the groups (Table 1(a)). The majority of additional joint symptoms with anastrozole occurred during the first 2 years of treatment. Significant risk factors for joint symptoms from multivariate analysis included prior use of hormone-replacement therapy, prior chemotherapy, geographic region (higher in North America, lower in the United Kingdom), BMI > 30, and positive hormone receptor status (Table 1(b)) [20]. The investigators noted that all of these factors are potentially linked with a greater decline in estrogen levels at the time of initiation of endocrine treatment.

## 4. Efficacy and Joint Symptoms in Adjuvant AI Trials

### 4.1. AIs Versus Tamoxifen

 Comparative studies have generally reported a higher incidence of both arthralgia and myalgia with AIs versus tamoxifen [2, 3, 21, 22, 25] (Table 2), although it should be remembered that AEs were not necessarily defined homogeneously between trials. Initial adjuvant therapy with AIs has been examined in the ATAC, BIG 1-98, and Tamoxifen Exemestane Adjuvant Multicenter (TEAM) trials. In terms of efficacy, both anastrozole and letrozole improved DFS over tamoxifen in patients with HR+ disease, whereas a significant benefit of exemestane in this setting has not yet been demonstrated [21]. It has been postulated that the near-complete suppression of estrogen levels achieved with the third-generation AIs relative to tamoxifen, ovarian ablation, or second-generation AIs may be one reason for an increased frequency of arthralgia/myalgia with AIs [26, 27]. Indeed, recent retrospective data from the ATAC trial suggest that joint symptoms in patients on endocrine therapy may be an important indicator of efficacy, as they are related to significant estrogen suppression with anastrozole. It was shown that the risk of breast cancer recurrence was lower in women with joint symptoms (hazard ratio [HR], 0.60; *P* < .0001) relative to those without joint symptoms [28]. These results held true regardless of treatment group. For anastrozole-treated patients with joint symptoms at 3 months, the HR was 0.65 (*P* = .001), and for tamoxifen-treated patients, the HR was 0.58 (*P* < .0001) [28]. These findings suggest that the emergence of joint symptoms with either AIs or tamoxifen could be an important predictor for lower recurrence risk. COSTART-defined joint symptoms, including arthralgia, arthritis, arthrosis, and joint disorder were reported by significantly more anastrozole-treated patients in the ATAC study at 68 months' followup (Table 2) [1]. Nearly half (46%) of patients reporting joint symptoms in ATAC, however, reported them as an exacerbation of preexisting conditions [29]. Most events (68% anastrozole and 59% tamoxifen) occurred within the first 24 months of therapy, and the peak occurrence was at 6 months (29% and 20%, resp.). Importantly, half of the patients whose joint symptoms resolved were symptom-free within 6 months, and 75% were free of symptoms within 18 months. Sixty percent received treatment for joint symptoms, with >90% of these patients managing their joint symptoms with nonsteroidal anti-inflammatory drugs (NSAIDs) alone or in combination with other analgesics. Serious joint symptoms were similar between the groups (10.6% versus 10.4%), and joint symptoms were rarely a cause of treatment withdrawal (2.1% versus 0.9%) [29]. The pathogenesis of carpal tunnel syndrome and its potential association with AI use is not fully understood, but hormonal factors may play a role, in view of its association with pregnancy and menopause, and its predominance in women [16, 30]. In the ATAC study, there was a significantly higher incidence of carpal tunnel syndrome with anastrozole versus tamoxifen, but the overall incidence was low (3% versus 1%; *P* < .0001) [25]. Initial adjuvant therapy with letrozole has also been associated with a higher incidence of joint symptoms compared with tamoxifen. The BIG 1-98 trial reported a significantly higher incidence of arthralgia, although the incidence of myalgia was not different between groups (Table 2) [2]. Most reported arthralgias and myalgias in both treatment groups in BIG 1-98 were grade 1 or 2. The incidence of carpal tunnel syndrome has not been reported in BIG 1-98.

An increased incidence of arthralgia and myalgia is not limited to the use of nonsteroidal AIs. Patients receiving the steroidal AI exemestane in the TEAM trial had a significantly higher incidence of arthralgia compared with those receiving tamoxifen (Table 2). A subanalysis from the TEAM trial also showed that, at 12 months, patients treated with exemestane (*n* = 808) had a significantly higher incidence of bone/muscle aches than did patients treated with tamoxifen (*n* = 806) (77% versus 70%, resp.; *P* < .0001) [31]. More than 60% of women reported mild, moderate, or severe bone and/or muscle aches at the time of study entry. In the IES, patients who switched to exemestane after 2−3 years of tamoxifen also had both a higher incidence of arthralgia (Table 2) and also carpal tunnel syndrome (2.8% versus 0.4%, resp.; *P* < .0001) compared with patients who remained on tamoxifen [3]. Switching to an AI following 2 years of prior tamoxifen treatment was also associated with an increase in arthralgia/bone pain in the Arimidex-Nolvadex 95 study (*N* = 979); more patients who switched to anastrozole from tamoxifen reported this symptom than did those continued on tamoxifen (Table 2) [23]. Higher rates of arthralgia/myalgia have also been seen in the extended adjuvant setting in postmenopausal women who have already received 5 years of prior adjuvant tamoxifen. In the extended adjuvant MA.17 trial, a significantly higher incidence of arthralgia and myalgia with letrozole relative to placebo was seen in women who had received a full course (4.5−6.0 years) of tamoxifen therapy (Table 2) [24]. The incidence of arthritis was not significantly different between the two groups (6% versus 5%; *P* = .07). 

Taken together, these studies suggest that the profound suppression of estrogen by AIs in postmenopausal women can result in an exacerbation of menopausal symptoms and the emergence of musculoskeletal AEs, both in the initial adjuvant setting and in those who have received prior tamoxifen treatment. Despite these findings, however, clinical trials of AIs have failed to find a significant worsening in QoL measures for patients on AIs relative to tamoxifen or placebo, suggesting no apparent measurable impact on day-to-day function and activity, even if patients were experiencing musculoskeletal symptoms while on AIs [32–35].

## 5. AI-Related Musculoskeletal Symptoms in Clinical Practice

Evidence from patient-perspective studies indicate that joint aches are among the most troublesome symptoms for patients taking AIs. In a large study of AI-related side effects (*N* = 612), the most commonly reported side effects were joint-related (joint pain, arthritis, and stiffness), and the AI discontinuation rate was 30%, with 84% of these patients discontinuing therapy primarily because of joint-related symptoms [12]. Another recent cross-sectional study examined the incidence of joint symptoms in postmenopausal women (*N* = 200) receiving AI treatment for breast cancer (64% anastrozole, 19% letrozole, 17% exemestane) [10]. These investigators found a 47% incidence of AI-related joint pain, while 23.5% reported a worsening of preexistent joint pain. Similarly, 44% of patients in this study reported AI-related joint stiffness, 26.5% reported new-onset joint stiffness, and 17.5% reported a worsening of baseline joint stiffness after starting AI treatment. Symptoms were considered severe in 20%−25% of the patients. A smaller study of patients outside of clinical trials receiving AIs (*N* = 56) found new or worsening symptoms in 61% of the patients, with a severity of 7.5 on a 10-point scale; notably, 30% rated their pain as 8−10, and the discontinuation rate was 20% for arthralgia [11]. There was no apparent relation between arthralgia and age or duration of AI use. Findings from these studies thus suggest a higher “real-life” occurrence of musculoskeletal symptoms with AI use than that observed in randomized adjuvant trials (between 4% and 35%), and identify these symptoms as important causes of treatment discontinuation; this can lead to a corresponding decrease in efficacy and a potential negative impact on breast cancer outcomes.

## 6. Managing AI-Induced Arthralgia

A temporal relationship between new-onset pain or the worsening of preexisting pain and the initiation of an AI makes the diagnosis of AI-related arthralgia or myalgia rather straightforward. On physical examination, there is absence of the inflammatory findings typical of arthritis [13]. Patients should be reassured that their symptoms are not due to permanent changes in the joints or joint destruction and are reversible upon AI withdrawal, that the exacerbation of joint symptoms may be a surrogate marker of the effectiveness of the hormonal therapy and may be associated with a reduced risk of breast cancer recurrence [28], and that the symptoms will likely improve with time. The strategies outlined below may be useful to consider in the management of joint symptoms.

### 6.1. Over-the-Counter (OTC) Pain Relievers

 OTC pain medications, including acetaminophen and ibuprofen, are effective therapies for minor arthralgia and myalgia symptoms and are appropriate initial therapies for symptoms of acute joint pain [36]. Many women receiving AI therapy may opt for these medications in lieu of discontinuing hormonal treatment. In a cross-sectional study of arthralgias secondary to AIs, a little more than half of the women with AI-induced arthralgias used pain medicines [10]. NSAIDs and acetaminophen were the most commonly used OTC medications (82%). A minority of women (7%) required more potent opiate medications for pain relief. Nearly 80% of patients taking oral medications reported moderate to complete relief of their arthralgia symptoms [10].

### 6.2. Switching to an Alternative Endocrine Therapy

 Switching between endocrine therapies may be another option for patients whose symptoms are troublesome, persistent, and/or not effectively managed with OTC pain medications. One such strategy involves switching to another AI. This option may be preferable for many patients, as the superior efficacy in preventing recurrence with AIs over tamoxifen is retained.

The impact of the nonsteroidal AIs (letrozole and anastrozole) on joint pain and stiffness was examined in a randomized adjuvant trial [37]. There were no significant differences in the frequency or grade of joint symptoms between letrozole and anastrozole; significantly fewer joint symptoms were reported with tamoxifen than with either letrozole (*P* = .0192) or anastrozole (*P* = .0126), and three quarters of patients who experienced joint symptoms while on an AI did not have the same problems on tamoxifen. Notably, more than half of the patients with symptoms on one AI did not have the same problems on the other AI, suggesting that switching therapies could improve symptoms for some patients [37]. In the 6-month prospective, open-label articular tolerance of letrozole (ATOLL) study, patients (*N* = 179) who experienced severe joint pain requiring discontinuation while on anastrozole were switched to letrozole [38]. Patients were initiated on letrozole following a 1-month washout period. At the end of the 6-month followup, 71.5% of the patients remained on letrozole electively, while 28.5% of the patients discontinued because of severe joint pain; the most commonly affected joints were the hands, knees, ankles, and spine. Patients had been on prior anastrozole for a mean of 14.6 months, and the duration of prior anastrozole was the only significant predictor of letrozole discontinuation identified, such that a shorter duration of prior anastrozole was associated with a higher risk of letrozole discontinuation (*P* = .04). Age, menopause duration, sociodemographic status, and previous osteoarthritis had no impact [38]. Fewer women overall reported arthralgia after switching (87% versus 74%), and this was observed uniformly across all affected joints. Brief Pain Inventory scores also decreased significantly after the switch to letrozole (*P* < .001), and QoL measures were significantly improved [38]. These results suggest that in this setting, switching from anastrozole to letrozole allows a sizable proportion of patients to continue to receive the benefits of more effective AI therapy while alleviating joint symptoms.

### 6.3. Nonpharmacologic Approaches

 Simple, low-cost, or no-cost lifestyle changes have been suggested to improve women's bone health and reduce the risk for osteoporosis. These changes include increased physical activity, load-bearing exercises, smoking cessation, and the avoidance of excessive alcohol and caffeine [39]. The same measures may be useful to women dealing with AI-associated arthralgia and myalgia, although there are no conclusive data to prove their effectiveness. Other nonpharmacologic approaches include weight loss, physical or occupational therapy, and targeted heat therapy (e.g., heat packs or hot showers) [40].

### 6.4. Glucosamine

 One small series (*N* = 56) has reported the efficacy of glucosamine in 15% of patients with AI-related arthralgia and/or bone pain [11]. Glucosamine may be a useful alternative therapy to reduce pain and improve function in patients with osteoarthritis, although there are no definitive data or recommendations regarding dosing or the efficacy of one preparation over another [41]. Moreover, we are unaware of any controlled trials of glucosamine specifically for the treatment of patients with AI-induced arthralgia.

### 6.5. Acupuncture

 A single-arm feasibility trial has been conducted to determine whether electroacupuncture of affected joints (twice weekly for 2 weeks, followed by once weekly for 6 weeks) could reduce pain and stiffness in patients with AI-related arthralgia [42]. Using the brief joint symptom inventory assessment, significant improvement from baseline in joint pain severity (*P* < .0001), joint stiffness (*P* < .0001), and joint functional symptom interference (*P* = .0001) were observed with the intervention. The procedure was well-tolerated, and there was also significant improvement in fatigue (*P* = .005) and anxiety (*P* = .014) end points examined [42]. Although requiring confirmation in a randomized controlled trial, this study demonstrates the feasibility of this method to control AI-related arthralgia without discontinuating AI therapy [42].

### 6.6. Vitamin D Supplementation and Joint Pain from AIs

 Vitamin D deficiency is associated with a syndrome of musculoskeletal symptoms with generalized nonspecific musculoskeletal pain and stiffness, as well as impaired muscle strength and function that is similar to that induced by AI therapy. Hypovitaminosis D has been suggested as an underlying etiology in individuals with persistent, nonspecific musculoskeletal pain [43]. In addition to musculoskeletal symptoms, vitamin D deficiency has been implicated in accelerated bone loss in women with breast cancer receiving AI therapy [44]. A possible biologic explanation of AI-induced musculoskeletal symptoms and their improvement with vitamin D supplementation is that reduction in joint estrogen levels may unmask subclinical Vitamin D deficiency. Estrogen increases activity of 1-alpha hydroxylase responsible for conversion of 25OHD to the biologically active 1,25-dihydroxyvitamin D [45, 46]. In addition estrogen increases expression of the Vitamin D receptor and VDR gene via activation of ERK 1/2 signaling pathway [47, 48]. Increasing vitamin D substrate via higher doses may increase the active hormone 1,25-dihydroxyvitamin D with resultant reduction in joint symptoms.

We conducted a pilot study to assess the prevalence of suboptimal vitamin D levels in 60 women initiating adjuvant letrozole for breast cancer, and to determine whether supplementation with 50,000 IU of vitamin D3 weekly could reduce musculoskeletal symptoms in women with suboptimal vitamin D levels [49]. Baseline 25OHD levels were obtained, and women were started on letrozole. Four weeks later, women with baseline 25OHD levels ≤40 ng/mL were started on vitamin D3 supplementation of 50,000 IU per week. At week 16, after 12 weeks on high-dose vitamin D, 25OHD levels were measured. Disability from joint pain was assessed using the validated Health Assessment Questionnaire (HAQ) II at baseline and at week 16. We found that at baseline, 63% of women exhibited vitamin D deficiency (<20 ng/mL) or insufficiency (20–31 ng/mL). 25OHD levels > 40 ng/mL were achieved in all 42 subjects who received 12 weeks of supplementation with 50,000 IU vitamin D3 weekly, with no adverse effects. Our results also showed that 50,000 IU of vitamin D3, when given weekly to postmenopausal women starting adjuvant AI therapy, resulted in clinically significant improvement in disability from joint symptoms. After 16 weeks of letrozole, HAQ II scores were better in women whose 25OHD levels were above versus below the median of 66 ng/mL (*P* = .008, Mann-Whitney test). In addition, more women with 25OHD levels > 66 ng/mL (median level) reported no disability from joint pain than did women with levels < 66 ng/mL (52 versus 19%; *P* = .026). We concluded that vitamin D deficiency and insufficiency were prevalent in postmenopausal women initiating adjuvant AI. Vitamin D3 supplementation with 50,000 IU per week was safe, significantly increased 25OHD levels, and reduced disability from AI-induced arthralgias. A randomized trial to confirm suggestions of a vitamin D protective effect against AI-associated disability from joint pain is underway (see clinicaltrials.gov NCT00867217). Interestingly, our results contrast with those of Dizdar et al., who found significantly higher levels of 25OHD in patients receiving AIs with arthralgia symptoms versus those without symptoms (*P* = .001) or control patients not receiving AIs (*P* = .005) [16]. Differences in supplementation among the groups studied may have accounted for these findings, however, and no conclusions regarding the impact on arthralgia were made in the study. 

## 7. Conclusions

Musculoskeletal symptoms are frequent in women treated with AIs and may adversely affect compliance with this class of medications. The major clinical trials of the AIs have been inconsistent in reporting musculoskeletal events and have likely underestimated the incidence, in view of the findings from clinical practice. In addition, the evaluation of joint symptoms remains largely subjective, and there is a need for more standardized assessments. Further research is needed to understand the exact mechanism and pathophysiology of this side effect. To date, there are no proven treatment interventions. Early data on vitamin D supplementation look promising, and results from a larger, ongoing phase III trial are awaited.

## Figures and Tables

**Table:** (a)

	Anastrozole *N* = 2,698	Tamoxifen *N* = 2,735	OR (95% CI)
Overall	35.2%	30.3%	1.25 (1.11–1.40)
Mild	19.2%	17.3%	1.14 (0.99–1.31)
Moderate	13.6%	10.8%	1.29 (1.09–1.53)
Severe	2.4%	2.2%	1.06 (0.73–1.54)

**Table:** (b)

	OR (95% CI)	*P* Value
Anastrozole versus tamoxifen	1.26 (1.12–1.42)	<.0001
Previous hormone replacement therapy versus none	1.53 (1.35–1.74)	<.0001
Chemotherapy versus none	1.24 (1.07–1.43)	.004

Hormone receptor status		
Negative versus positive	0.78 (0.62–0.98)^a^	.03
Unknown versus positive	0.76 (0.59–0.97)^a^	.028

Region		
UK versus rest of world	1.20 (1.02–1.40)	.025
North America versus rest of world	2.09 (1.79–2.43)	<.0001
Body mass index >30	1.33 (1.14–1.55)	<.0001

^a^ Negative predictor.

CI: confidence interval

**Table 2:** Incidence of arthralgia and myalgia in postmenopausal women with early breast cancer as reported from phase III trials of third-generation aromatase inhibitors in the adjuvant setting.

Phase 3 trial	Median followup (months)	Arthralgia (%)	Myalgia (%)
ATAC [1]	68	35.6% ANA versus 29.4% TAM^a^; *P* < .0001	NR
BIG 1-98 [2]	25.8	20.3% LET versus 12.3% TAM^b^; *P* < .001	6.4% LET versus 6.1% TAM; *P *=.61
BIG 1-98 [8]	51	20.0% LET versus 13.5% TAM; *P* < .001	7.1% LET versus 6.1% TAM; *P* = .19
TEAM [21]	33	17.9% EXE versus 9.2% TAM; *P* < .001	NR
IES [3]	55.7	20.8% EXE versus 15.1% TAM^b^; *P* < .0001	25.7% EXE versus 20.3% TAM^c^; *P* < .0001
ABCSG 8/ARNO 95 [22]	28	19% ANA versus 16% TAM^d^; *P* = .0546	NR
ARNO 95 [23]	30.1	11.7% ANA versus 4.9% TAM^e^	NR
MA.17 [24]	30	25% LET versus 21% PLA^b^; *P* < .001	15% LET versus 12% PLA; *P* = .004

ABCSG: austrian breast and colorectal cancer study group; ANA: anastrozole; ARNO: arimidex-nolvadex; ATAC: arimidex tamoxifen alone or in combination; BIG: breast international group; EXE: exemestane; IES: intergroup exemestane study; LET: letrozole; PLA: placebo; TAM: tamoxifen; TEAM: tamoxifen exemestane adjuvant multicenter; NR: not reported.

^a^Grade not specified.

^b^All grades.

## References

[1] Howell A, Cuzick J, Baum M (2005). Results of the ATAC (Arimidex, Tamoxifen, Alone or in Combination) trial after completion of 5 years' adjuvant treatment for breast cancer. *The Lancet*.

[2] Thürlimann B, Keshaviah A, Coates AS (2005). A comparison of letrozole and tamoxifen in postmenopausal women with early breast cancer. *The New England Journal of Medicine*.

[3] Coombes R, Kilburn L, Snowdon C (2007). Survival and safety of exemestane versus tamoxifen after 2-3 years’ tamoxifen treatment (Intergroup Exemestane Study): a randomised controlled trial. *The Lancet*.

[4] Mouridsen H, Giobbie-Hurder A, Goldhirsch A (2009). Letrozole therapy alone or in sequence with tamoxifen in women with breast cancer. *The New England Journal of Medicine*.

[5] Aiello EJ, Buist DSM, Wagner EH (2008). Diffusion of aromatase inhibitors for breast cancer therapy between 1996 and 2003 in the Cancer Research Network. *Breast Cancer Research and Treatment*.

[6] Braithwaite RS, Chlebowski RT, Lau J, George S, Hess R, Col NF (2003). Meta-analysis of vascular and neoplastic events associated with tamoxifen. *Journal of General Internal Medicine*.

[7] Forbes JF, Cuzick J, Buzdar A (2008). Effect of anastrozole and tamoxifen as adjuvant treatment for early-stage breast cancer: 100-month analysis of the ATAC trial. *The Lancet Oncology*.

[8] Coates AS, Keshaviah A, Thürlimann B (2007). Five years of letrozole compared with tamoxifen as initial adjuvant therapy for postmenopausal women with endocrine-responsive early breast cancer: update of study BIG 1-98. *Journal of Clinical Oncology*.

[9] Garreau JR, DeLaMelena T, Walts D, Karamlou K, Johnson N (2006). Side effects of aromatase inhibitors versus tamoxifen: the patients’ perspective. *American Journal of Surgery*.

[10] Crew KD, Greenlee H, Capodice J (2007). Prevalence of joint symptoms in postmenopausal women taking aromatase inhibitors for early-stage breast cancer. *Journal of Clinical Oncology*.

[11] Presant CA, Bosserman L, Young T (2007). Aromatase inhibitor-associated arthralgia and/or bone pain: frequency and characterization in non-clinical trial patients. *Clinical Breast Cancer*.

[12] Salgado BA, Zivian MT (2006). Aromatase inhibitors: side effects reported by 622 women. *Breast Cancer Research and Treatment*.

[13] Burstein HJ (2007). Aromatase inhibitor-associated arthralgia syndrome. *Breast*.

[14] Morales L, Pans S, Paridaens R (2007). Debilitating musculoskeletal pain and stiffness with letrozole and exemestane: associated tenosynovial changes on magnetic resonance imaging. *Breast Cancer Research and Treatment*.

[15] Morales L, Pans S, Verschueren K (2008). Prospective study to assess short-term intra-articular and tenosynovial changes in the aromatase inhibitor-associated arthralgia syndrome. *Journal of Clinical Oncology*.

[16] Dizdar O, Özçakar L, Malas FU (2009). Sonographic and electrodiagnostic evaluations in patients with aromatase inhibitor-related arthralgia. *Journal of Clinical Oncology*.

[17] Xu J, Bartoces M, Neale AV, Dailey RK, Northrup J, Schwartz KL (2005). Natural history of menopause symptoms in primary care patients: a MetroNet study. *Journal of the American Board of Family Practice*.

[18] Fuh J-L, Wang S-J, Lee S-J, Lu S-R, Juang K-D (2003). Quality of life and menopausal transition for middle-aged women on Kinmen island. *Quality of Life Research*.

[19] Bingefors K, Isacson D (2004). Epidemiology, co-morbidity, and impact on health-related quality of life of self-reported headache and musculoskeletal pain—a gender perspective. *European Journal of Pain*.

[20] Sestak I, Cuzick J, Sapunar F (2008). Risk factors for joint symptoms in patients enrolled in the ATAC trial: a retrospective, exploratory analysis. *The Lancet Oncology*.

[21] Jones SE, Seynaeve C, Hasenburg A (2009). Results of the first planned analysis of the TEAM (tamoxifen exemestane adjuvant multinational) prospective randomized phase III trial in hormone sensitive postmenopausal early breast cancer. *Cancer Research*.

[22] Jakesz R, Jonat W, Gnant M (2005). Switching of postmenopausal women with endocrine-responsive early breast cancer to anastrozole after 2 years’ adjuvant tamoxifen: combined results of ABCSG trial 8 and ARNO 95 trial. *The Lancet*.

[23] Kaufmann M, Jonat W, Hilfrich J (2007). Improved overall survival in postmenopausal women with early breast cancer after anastrozole initiated after treatment with tamoxifen compared with continued tamoxifen: the ARNO 95 study. *Journal of Clinical Oncology*.

[24] Goss PE, Ingle JN, Martino S (2005). Randomized trial of letrozole following tamoxifen as extended adjuvant therapy in receptor-positive breast cancer: updated findings from NCIC CTG MA.17. *Journal of the National Cancer Institute*.

[25] Buzdar A, Howell A, Cuzick J (2006). Arimidex, Tamoxifen, Alone or in Combination Trialists' Group. Comprehensive side-effect profile of anastrozole and tamoxifen as adjuvant treatment for early-stage breast cancer: long-term safety analysis of the ATAC trial. *The Lancet Oncology*.

[26] Donnellan PP, Douglas SL, Cameron DA, Leonard RCF (2001). Aromatase inhibitors and arthralgia. *Journal of Clinical Oncology*.

[27] Mackey J, Gelmon K (2007). Adjuvant aromatase inhibitors in breast cancer therapy: significance of musculoskeletal complications. *Current Opinion in Oncology*.

[28] Cuzick J, Sestak I, Cella D, Fallowfield L (2008). Treatment-emergent endocrine symptoms and the risk of breast cancer recurrence: a retrospective analysis of the ATAC trial. *The Lancet Oncology*.

[29] Buzdar AU (2006). Clinical features of joint symptoms observed in the ‘Arimidex’, Tamoxifen, alone or in combination (ATAC) trial. *Journal of Clinical Oncology*.

[30] Aroori S, Spence RAJ (2008). Carpal tunnel syndrome. *Ulster Medical Journal*.

[31] Jones SE, Cantrell J, Vukelja S (2007). Comparison of menopausal symptoms during the first year of adjuvant therapy with either exemestane or tamoxifen in early breast cancer: report of a tamoxifen exemestane adjuvant multicenter trial substudy. *Journal of Clinical Oncology*.

[32] Whelan TJ, Pritchard KI, Arteaga C, Come S, Ingle J (2006). Managing patients on endocrine therapy: focus on quality-of-life issues. *Clinical Cancer Research*.

[33] Whelan TJ, Goss PE, Ingle JN (2005). Assessment of quality of life in MA. 17: a randomized, placebo-controlled trial of letrozole after 5 years of tamoxifen in postmenopausal women. *Journal of Clinical Oncology*.

[34] Fallowfield L, Cella A, Cuzick J, Francis S, Locker G, Howell A (2004). Quality of life of postmenopausal women in the Arimidex Tamoxifen, Alone or in Combination (ATAC) adjuvant breast cancer trial. *Journal of Clinical Oncology*.

[35] Fallowfield LJ, Bliss JM, Porter LS (2006). Quality of life in the intergroup exemestane study: a randomized trial of exemestane versus continued tamoxifen after 2 to 3 years of tamoxifen in postmenopausal women with primary breast cancer. *Journal of Clinical Oncology*.

[36] Palmer T, Toombs JD (2004). Managing joint pain in primary care. *Journal of the American Board of Family Practice*.

[37] Renshaw L, McHugh RGN, Williams L (2007). Comparison of joint problems reported by patients in a randomized adjuvant trial of anastrozole and letrozole. *Breast Cancer Research and Treatment*.

[38] Briot K, Tubiana-Hulin M, Bastit L, Kloos I, Roux C (2010). Effect of a switch of aromatase inhibitors on musculoskeletal symptoms in postmenopausal women with hormone-receptor-positive breast cancer: the ATOLL (articular tolerance of letrozole) study. *Breast Cancer Research and Treatment*.

[39] Moyad MA (2002). Complementary therapies for reducing the risk of osteoporosis in patients receiving luteinizing hormone-releasing hormone treatment/orchiectomy for prostate cancer: a review and assessment of the need for more research. *Urology*.

[40] Cella D, Fallowfield LJ (2008). Recognition and management of treatment-related side effects for breast cancer patients receiving adjuvant endocrine therapy. *Breast Cancer Research and Treatment*.

[41] Fox BA, Schmitz ED, Wallace R (2006). FPIN’s clinical inquiries. Glucosamine and chondroitin for osteoarthritis. *American Family Physician*.

[42] Mao JJ, Bruner DW, Stricker C (2009). Feasibility trial of electroacupuncture for aromatase inhibitor-related arthralgia in breast cancer survivors. *Integrative Cancer Therapies*.

[43] Plotnikoff GA, Quigley JM (2003). Prevalence of severe hypovitaminosis D in patients with persistent, nonspecific musculoskeletal pain. *Mayo Clinic Proceedings*.

[44] Lønning PE (2006). Bone safety of aromatase inhibitors versus tamoxifen. *International Journal of Gynecological Cancer*.

[45] Buchanan JR, Santen R, Cauffman S, Cavaliere A, Greer RB, Demers LM (1986). The effect of endogenous estrogen fluctuation on metabolism of 25-hydroxyvitamin D. *Calcified Tissue International*.

[46] Cheema C, Grant BF, Marcus R (1989). Effects of estrogen on circulating ’free’ and total 1,25-dihydroxyvitamin D and on the parathyroid-vitamin D axis in postmenopausal women. *Journal of Clinical Investigation*.

[47] Gilad LA, Bresler T, Gnainsky J, Smirnoff P, Schwartz B (2005). Regulation of vitamin D receptor expression via estrogen-induced activation of the ERK 1/2 signaling pathway in colon and breast cancer cells. *Journal of Endocrinology*.

[48] Escaleira MTF, Sonohara S, Brentani MM (1993). Sex steroids induced up-regulation of 1,25-(OH)_2_ vitamin D_3_ receptors in T 47D breast cancer cells. *Journal of Steroid Biochemistry and Molecular Biology*.

[49] Khan QJ, Reddy PS, Kimler BF (2010). Effect of vitamin D supplementation on serum 25-hydroxy vitamin D levels, joint pain, and fatigue in women starting adjuvant letrozole treatment for breast cancer. *Breast Cancer Research and Treatment*.

